# A nomogram model to individually predict prognosis for esophageal cancer with synchronous pulmonary metastasis

**DOI:** 10.3389/fonc.2022.956738

**Published:** 2023-01-04

**Authors:** Xin-yao Zhang, Qi-yuan Lv, Chang-lin Zou

**Affiliations:** ^1^ Department of Pediatrics, the First Affiliated Hospital of Wenzhou Medical University, Wenzhou, Zhejiang, China; ^2^ School of Public Health and Management, Wenzhou Medical University, Wenzhou, Zhejiang, China; ^3^ Department of Radiotherapy, the First Affiliated Hospital of Wenzhou Medical University, Wenzhou, Zhejiang, China

**Keywords:** esophageal cancer (EC), pulmonary metastasis, nomogram, prognostic factors, cancer-specific survival, chemotherapy

## Abstract

**Background:**

Esophageal cancer (EC) is a life−threatening disease worldwide. The prognosis of EC patients with synchronous pulmonary metastasis (PM) is unfavorable, but few tools are available to predict the clinical outcomes and prognosis of these patients. This study aimed to construct a nomogram model for the prognosis of EC patients with synchronous PM.

**Methods:**

From the Surveillance, Epidemiology, and End Results database, we selected 431 EC patients diagnosed with synchronous PM. These cases were randomized into a training cohort (303 patients) and a validation cohort (128 patients). Univariate and multivariate Cox regression analyses, along with the Kaplan-Meier method, were used to estimate the prognosis and cancer-specific survival (CSS) among two cohorts. Relative factors of prognosis in the training cohort were selected to develop a nomogram model which was verified on both cohorts by plotting the receiver operating characteristic (ROC) curves as well as the calibration curves. A risk classification assessment was completed to evaluate the CSS of different groups using the Kaplan-Meier method.

**Results:**

The nomogram model contained four risk factors, including T stage, bone metastasis, liver metastasis, and chemotherapy. The 6-, 12- and 18-month CSS were 55.1%, 26.7%, and 5.9% and the areas under the ROC curve (AUC) were 0.818, 0.781, and 0.762 in the training cohort. Likewise, the AUC values were 0.731, 0.764, and 0.746 in the validation cohort. The calibration curves showed excellent agreement both in the training and validation cohorts. There was a substantial difference in the CSS between the high-risk and low-risk groups (P<0.01).

**Conclusion:**

The nomogram model serves as a predictive tool for EC patients with synchronous PM, which would be utilized to estimate the individualized CSS and guide therapeutic decisions.

## Introduction

Esophageal cancer (EC) is a commonly diagnosed malignant tumor, ranking seventh and sixth respectively in terms of incidence and mortality (1). Indeed, over half of the EC patients are diagnosed with metastatic or unresectable disease at their first visit (2, 3). Distant lymph nodes, lung, and liver are the most common sites for EC metastases (4, 5). A recent study reported that 50% of EC patients could develop pulmonary metastases (PM) (6). Therefore, a prognosis evaluation is required for therapy and follow-up.

The prognosis of EC patients with synchronous PM is notoriously unfavorable, but few reports describe the cancer-specific survival (CSS) of these patients. Although some prior clinical studies reported that surgical resection and stereotactic body radiotherapy for PM from EC could be an option of personalized treatment (7–9), there is still little evidence about the standard treatment of palliative regimen for EC patients with synchronous PM (10). As a result, the majority of current therapies are based on clinical experience and literature, which means that no accessible method is available to predict the prognosis of these patients.

The nomogram, designed for an individual patient to predict mortality risks in a variety of diseases, is a widely used graphical prediction tool (11). It contains both pathological factors and clinical risk factors, such as metastatic sites, T stage, and treatments (12). This tool could provide clinicians with the survivability of patients and therefore assist them in developing better treatment strategies, such as clinical trials and hospice care. Hence, this study, through the clinical and pathological information from the SEER database, sought to establish a nomogram model for a personalized assessment of EC patients with synchronous PM.

## Materials & methods

### Data extraction

Patient data were extracted from the Surveillance, Epidemiology, and End Results (SEER) database, which serves as a population-based cancer registry system summarizing data from fourteen states throughout the United States, accounting for almost 35% of the American population. Patients (i) diagnosed with EC between 2010 and 2015, (ii) confirmed to have pulmonary metastasis at initial diagnosis, and (iii) aged 18–100 years were enrolled in the study. And the patients (i) with multiple primary cancer, and (ii) with missing or incomplete data (such as metastatic sites, T stage, N stage, grade, primary tumor size, radiation, surgery, and race) were excluded. EC patients without other distant organ metastases, such as lung, liver, and brain metastases, were also excluded from this study. The primary tumor was confirmed histopathologically. The primary site was mainly determined by surgical resection. The pathological grade and type of esophageal cancer were obtained by further analysis of pathological specimens. Metastasis of the primary tumor depends on the pathology or imaging diagnosis. All patients’ clinical and pathological as well as demographic data were analysed retrospectively. Informed consent was not required due to the anonymization characteristic of the SEER database.

### Study population and follow-up

The CSS was defined as the time elapsed between diagnosis and EC-related death or termination of follow-up. Tumor variables were gathered to evaluate the prognostic influence on CSS, including demographic factors (age, race and sex), the tumor characteristics (primary site, grade, tumor size, pathological type, AJCC T stage, AJCC N stage, and primary tumor resection), extrapulmonary metastasis (bone, brain, and liver metastasis), and treatments (radiotherapy and chemotherapy). Based on the above factors, CSS was introduced using the Kaplan-Meier method and comparing subgroups with log-rank tests.

### Statistical analysis

To validate the reliability of the nomogram model, we randomly divided all investigated cases into training and validation cohorts in a 7:3 ratio. The training cohort was utilized to construct the nomogram model to predict the CSS of patients. Then the nomogram model was validated with the data both from the training and validation cohort. And the risk classification assessment was performed in these cohorts respectively.

To minimize selection bias, we select potential prognostic factors with P<0.01 that were analyzed by Kaplan-Meier as an additional research object. Then, these factors (excluded radiotherapy) were subjected to multivariate analysis through the Cox regression model. Based on the above factors (after subsequent selection), the nomogram was constructed to evaluate the CSS in the training cohort. Receiver operating characteristic (ROC) curves were applied to evaluate the predictive effectiveness of this nomogram for 6-, 12-, or 18-month CSS. Based on the Cox model, calibration curves were drawn to evaluate the reliability of the nomogram. The patients, in evaluating calibration, were divided equally into 3 subgroups of size, and bootstrap-corrected CSS rates, according to 1000 bootstrap samples, were calculated by averaging the Kaplan-Meier estimates. Additionally, the risk classification assessment was performed using the survival package in the R language software for risk scoring. We calculated a comprehensive risk score for each sample based on individual factors from the multivariate Cox regression analysis. According to the median value of the score, the samples were divided into high-risk group and low-risk group, and CSS of the two groups was analyzed.

For independent model validation, the total points for each patient in the validation cohort were calculated in light of the produced nomogram. Then, utilizing the total points as a factor, the Cox regression in the validation cohort was performed, and therefore the ROC curve and the calibration curve were constructed according to the regression analysis. All analyses were carried out using R language (version 3.6.3) and SPSS version 26.0. It was considered statistically significant when P-value is less than 0.05.

## Results

### Patient characteristics

**Figure 1:**
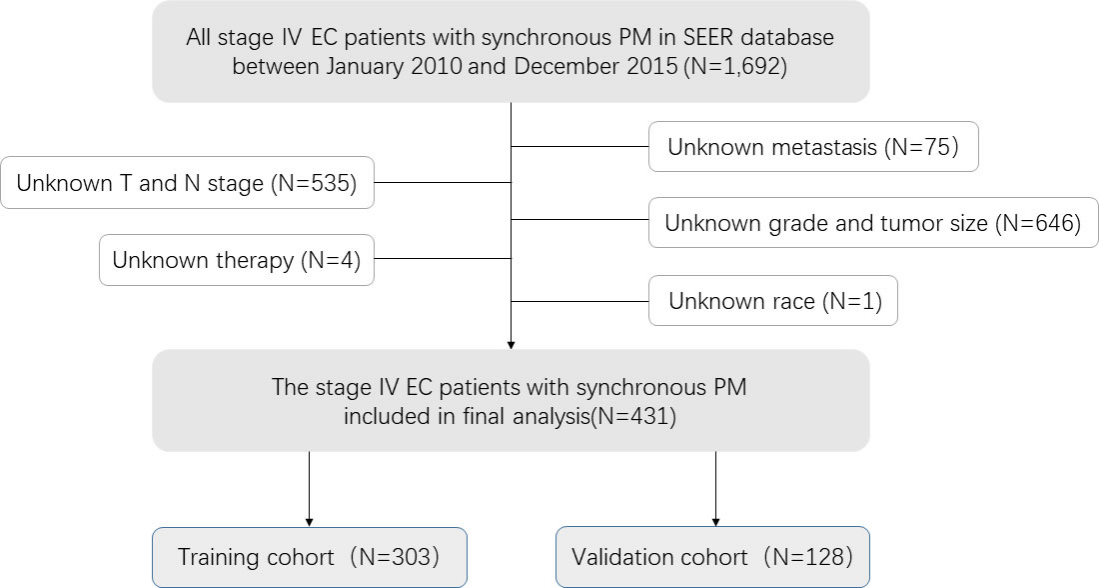
Analytical cohort and exclusion criteria for esophageal cancer patients with synchronous pulmonary metastasis.

Through preliminary data extraction, 1692 single EC patients with synchronous pulmonary metastases were found in the SEER database. Following the further screening, 431 Stage IV EC patients with synchronous PM were finally selected in the study cohort (**Figure 1**). Then we partitioned our patients randomly into training (70%) and validation cohort (30%) by R package “caret”, whose clinicopathological features were listed in **Table 1**. Indeed, the training cohort included 303 patients, and the validation cohort comprised 128 patients. The Chi-square test was performed on a single clinicopathological factor at both two study cohorts, and it was found that there was no statistical difference except for race factor and lymph node stage factor. Thus, the reliability of the results was assured. As shown in **Table 1**, training cohort patients with age≥65 (50.2%), white (81.5%), male (83.5%), abdominal or lower primary site (58.7%), high tumor grade (58.1%), tumor size≤0.5<1 cm (51.2%), adenocarcinoma (55.4%), T1 stage (32.7%), T4 stage (36.3%), and N1 (60.4%) had higher proportion. Some EC patients with synchronous PM had concurrent bone metastases (23.4%), brain metastases (6.9%), and liver metastases (39.6%). As a result, only a few patients received surgical therapy for primary tumors (3.5%). Most patients were treated with radiotherapy (45.9%) and chemotherapy (62.0%).

**Table 1:** Baseline clinicopathological characteristics and treatment experience of all patients.

Characteristics	All cohorts	Training cohort	Validation cohort	p value^*^
Age, years				0.907
<65	214(49.7)	151(49.8)	63(49.2)	
≥65	217(50.3)	152(50.2)	65(50.8)	
Race				0.001
other	32(7.4)	22(7.3)	10(7.8)	
black	66(15.3)	34(11.2)	32(25.0)	
white	333(77.3)	247(81.5)	86(67.2)	
Sex				0.668
female	69(16.0)	50(16.5)	19(14.8)	
male	362(84.0)	253(83.5)	109(85.2)	
Primary site				0.400
Cervical/upper	28(6.5)	19(6.3)	9(7.0)	
Thoracic/middle	97(22.5)	62(20.5)	35(27.3)	
Abdominal/lower	253(58.7)	185(61.1)	68(53.1)	
Overlapping/other	53(12.3)	37(12.2)	16(12.5)	
Tumor grade				0.515
GradeI,II	185(42.9)	127(41.9)	58(45.3)	
GradeIII,IV	246(57.1)	176(58.1)	70(54.7)	
Tumor size				0.838
<0.5cm	146(33.9)	100(33.0)	46(35.9)	
0.5≤ <1cm	218(50.6)	155(51.2)	63(49.2)	
≥1cm	67(15.5)	48(15.8)	19(14.8)	
Pathological type				0.436
squamous	160(37.1)	107(35.3)	53(41.4)	
adenocarcinoma	234(54.3)	168(55.4)	66(51.6)	
other	37(8.6)	28(9.2)	9(7.0)	
Stage T				0.170
T1	155(36.0)	99(32.7)	56(43.8)	
T2	14(3.2)	11(3.6)	3(2.3)	
T3	114(26.5)	83(27.4)	31(24.2)	
T4	148(34.3)	110(36.3)	38(29.7)	
Stage N				0.006
N0	99(23.0)	65(21.5)	34(26.6)	
N1	259(60.1)	183(60.4)	76(59.4)	
N2	41(9.5)	31(10.2)	10(7.8)	
N3	32(7.4)	24(7.9)	8(6.3)	
Bone metastasis				0.479
no	334(77.5)	232(76.6)	102(79.7)	
yes	97(22.5)	71(23.4)	26(20.3)	
Brain metastasis				0.228
no	405(94.0)	282(93.1)	123(96.1)	
yes	26(6.0)	21(6.9)	5(3.9)	
Liver metastasis				0.212
no	252(58.5)	183(60.4)	69(53.9)	
yes	179(41.5)	120(39.6)	59(46.1)	
Primary tumor resection				0.495
no surgery	419(97.2)	293(96.5)	126(98.4)	
surgery	12(2.8)	10(3.5)	2(1.6)	
Radiation				0.735
no	231(53.6)	164(54.1)	67(52.3)	
yes	200(46.4)	139(45.9)	61(47.7)	
Chemotherapy				0.261
no	171(39.7)	115(38.0)	56(43.8)	
yes	260(60.3)	188(62.0)	72(56.3)	

^*^ Chi-square test.

### Identification of predictive factors by univariate and multivariate analyses

The Cox proportional-hazards model was utilized to predict CSS in the training cohort by analyzing each variable. Univariate analyses presented that some factors such as primary site, tumor grade, T stage, N stage, bone metastasis, brain metastasis, liver metastasis, radiation, and chemotherapy were related to the prognosis of patients. Among these factors, stage T (index C=0.587) and chemotherapy (index C=0.687) were discriminated against to other factors (**Table 2A**), which may be significant predictors. To eliminate confounding factors, we eventually selected four factors in univariate analyses with a P-value<0.01 for further multivariate analyses. Consequently, factors such as T stage, bone metastasis, liver metastasis, and chemotherapy were contained in the predictive model and considered to be independent predictors of CSS (**Table 2B**).

**Table 2:** Univariate and multivariate analyses were performed to investigate each factor’s association with CSS.

(a) Univariate analyses	HR	95% CI of HR	P value	C-index
Age, years				
≥65 versus <65	0.959	0.754-1.220	0.735	0.495
Race				
black versus other	0.810	0.452-1.453	0.480	0.507
white versus other	0.886	0.560-1.401	0.605	
Sex				
male versus female	0.926	0.672-1.276	0.638	0.503
Primary site				
Thoracic/middle versus Cervical/upper	1.155	0.6662-2.002	0.608	0.532
Abdominal/lower versus Cervical/upper	1.285	0.7771-2.124	0.329	
Overlapping/other versus Cervical/upper	1.474	0.8232-2.638	0.192	
Tumor grade				
GradeIII,IV versus GradeI,II	1.295	1.013-1.656	0.039	0.537
Tumor size				
0.5≤ <1cm versus <0.5cm	0.925	0.707-1.210	0.568	0.541
≥1cm versus <0.5cm	1.178	0.814-1.705	0.384	
Pathological type				
adenocarcinoma versus squamous	0.920	0.710-1.194	0.533	0.525
other versus squamous	1.188	0.758-1.862	0.452	
Stage T				
T2 versus T1	0.455	0.229-0.904	0.025	0.587
T3 versus T1	0.566	0.413-0.777	0.000	
T4 versus T1	0.999	0.752-1.329	0.997	
Stage N				
N1 versus N0	0.814	0.607-1.092	0.170	0.526
N2 versus N0	0.822	0.523-1.293	0.396	
N3 versus N0	0.917	0.553-1.521	0.737	
Bone metastasis				
yes versus no	1.533	1.157-2.032	0.003	0.532
Brain metastasis				
yes versus no	1.793	1.120-2.870	0.015	0.522
Liver metastasis				
yes versus no	1.479	1.158-1.889	0.002	0.557
Primary tumor resection				
surgery versus no surgery	0.739	0.380-1.439	0.370	0.505
Radiation				
yes versus no	0.728	0.572-0.927	0.010	0.561
Chemotherapy				
yes versus no	0.206	0.157-0.271	0.000	0.687

(b) Multivariate analyses	HR	95% CI of HR	P value	C-index
				0.747
Stage T				
T2 versus T1	0.330	0.164-0.664	0.002	
T3 versus T1	0.756	0.547-1.047	0.092	
T4 versus T1	1.200	0.896-1.609	0.222	
Bone metastasis				
yes versus no	1.447	1.086-1.929	0.012	
Liver metastasis				
yes versus no	1.638	1.270-2.113	0.000	
Chemotherapy				
yes versus no	0.172	0.128-0.232	0.000	

### CSS analysis

**Figure 2:**
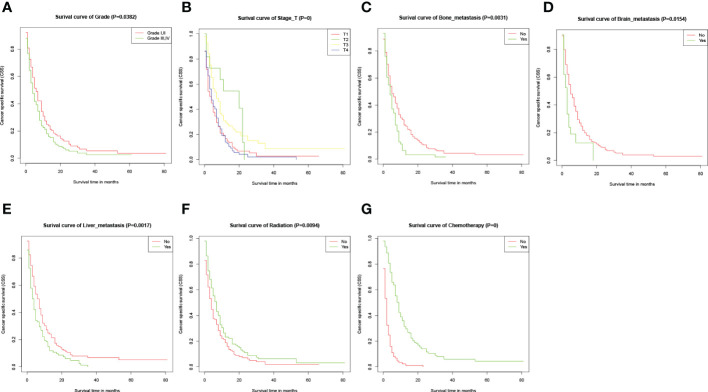
Kaplan-Meier CSS curves for several potential variables with P<0.05. **(A-G)** Kaplan-Meier curve of CSS based on grade, T-stage, bone metastasis, brain metastasis, liver metastasis, radiation, and chemotherapy respectively. The P-values are from a log-rank test for the comparison of the Kaplan-Meier curves.

At the time of the analysis, a total of 267 (88.12%) patients died of esophageal cancer within the training cohort, with a median CSS about 4 months. The 6-, 12- and 18-month CSS were 55.1%, 26.7%, and 5.9% in this cohort. Kaplan-Meier analysis was performed for each potential prognostic variable using the “survival package” from the R software. These factors included age (P=0.7351), race (P=0.4725), sex (P=0.6297), primary site (P=0.1361), tumor grade (P=0.0382), tumor size (P=0.16), pathological type (P=0.22), T stage (P=0), N stage (P=0.15), bone metastasis (P=0.0031), brain metastasis (P=0.0154), liver metastasis (P=0.0017), primary tumor resection (P=0.3653), radiotherapy (P=0.0094) and chemotherapy (P=0). And the Kaplan-Meier curves for certain variables with P values less than 0.05 were shown in **Figure 2**.

### Establishment and verification of the nomogram model

**Figure 3:**
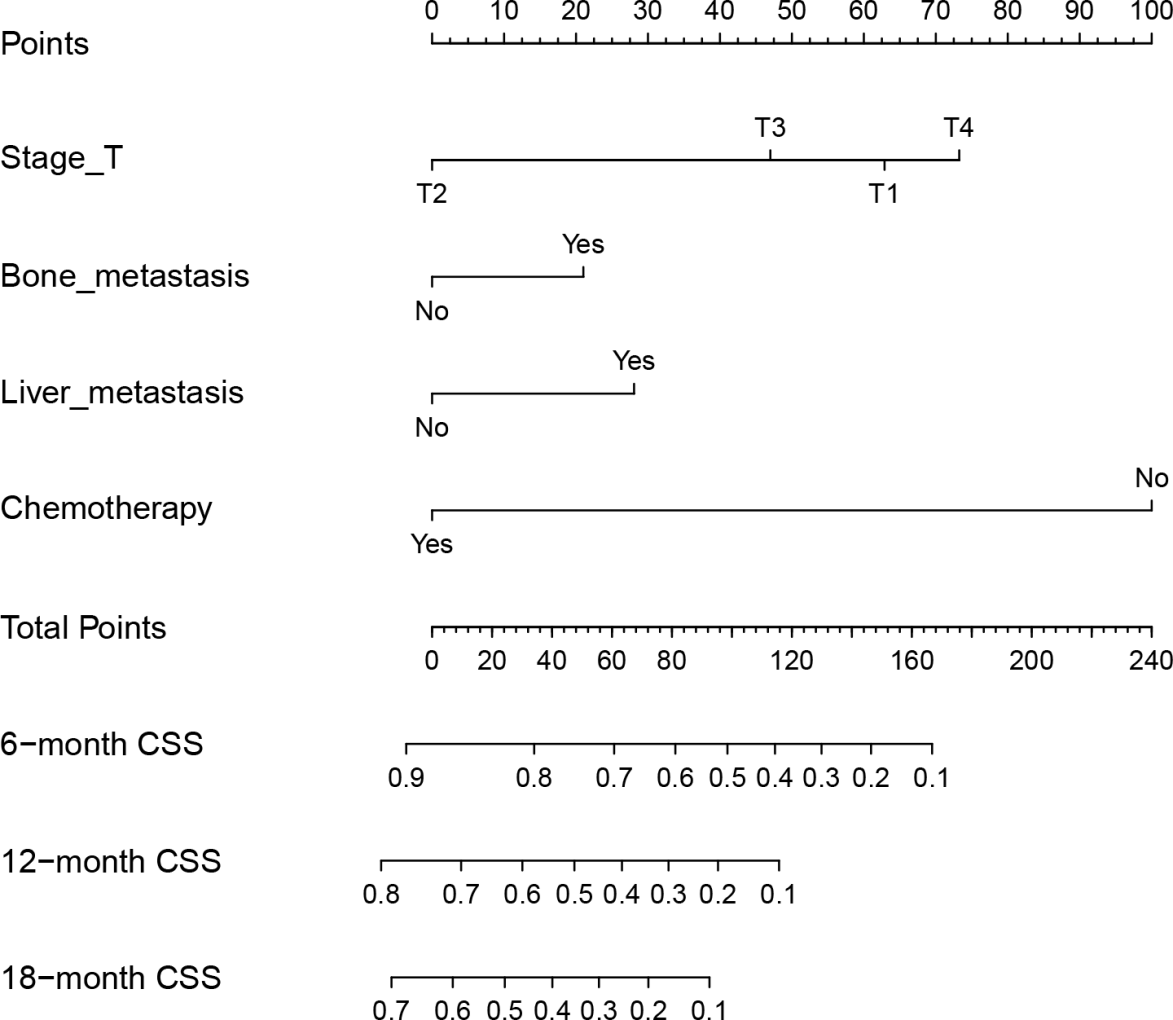
Nomogram for predicting the 6-, 12- and 18-month CSS of EC patients with synchronous PM. Nomogram used by totaling points identified at top scale for each of four independent variables (T stage, bone metastasis, liver metastasis, and chemotherapy). This summed point score then identified on total point scale to identify 6-, 12- and 18-month CSS.

**Figure 4:**
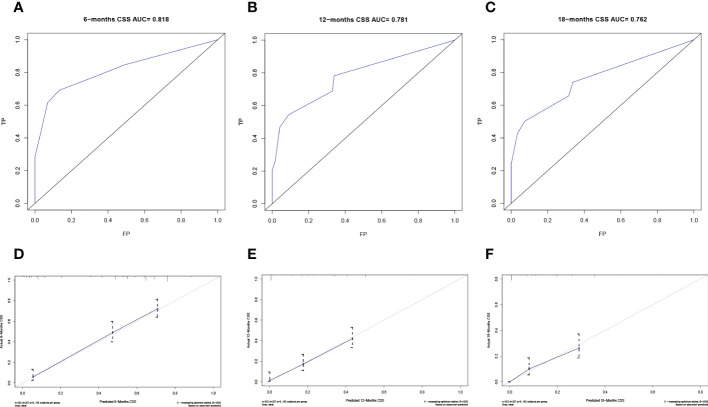
Verification of the nomogram in the training cohort. The ROC curve **(A-C)** and calibration curve **(D-F)** of the nomogram for the 6-, 12-, and 18-month CSS.

**Figure 5:**
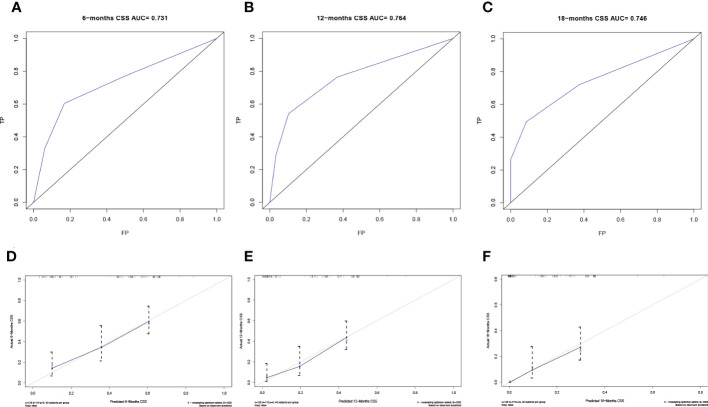
Verification of the nomogram in the validation cohort. The ROC curve **(A-C)** and calibration curve **(D-F)** of the nomogram for the 6-, 12-, and 18-month CSS.

The predictive model was visualized by the nomogram (**Figure 3**) and verified by training and validation cohorts. After analyzing the data from the training cohort, the C-index of the nomogram was 0.747, which means that the model has a well-distinguishing capability. Likewise, the ROC curve for the nomogram to predict the 6-, 12-, and 18-month CSS rates were presented in **Figures 4A-C**, and the area under ROC curve (AUC) values were 0.818, 0.781, and 0.762 respectively. In light of the data from the validation cohort, the corresponding ROC curve was shown in **Figures 5A-C**, and the AUC values were 0.731, 0.764, and 0.746 respectively. Besides, as the calibration curves shown in **Figures 4D-F** and **Figures 5D-F**, the probability CSS of 6-, 12-, and 18-month between model forecast and actual observation showed excellent agreement both in the training cohort and the validation cohort. The results not only suggested the satisfactory potential clinical effect of this model but also estimated the approximate survival time of patients with advanced esophageal cancer.

### Risk classification assessment

**Figure 6:**
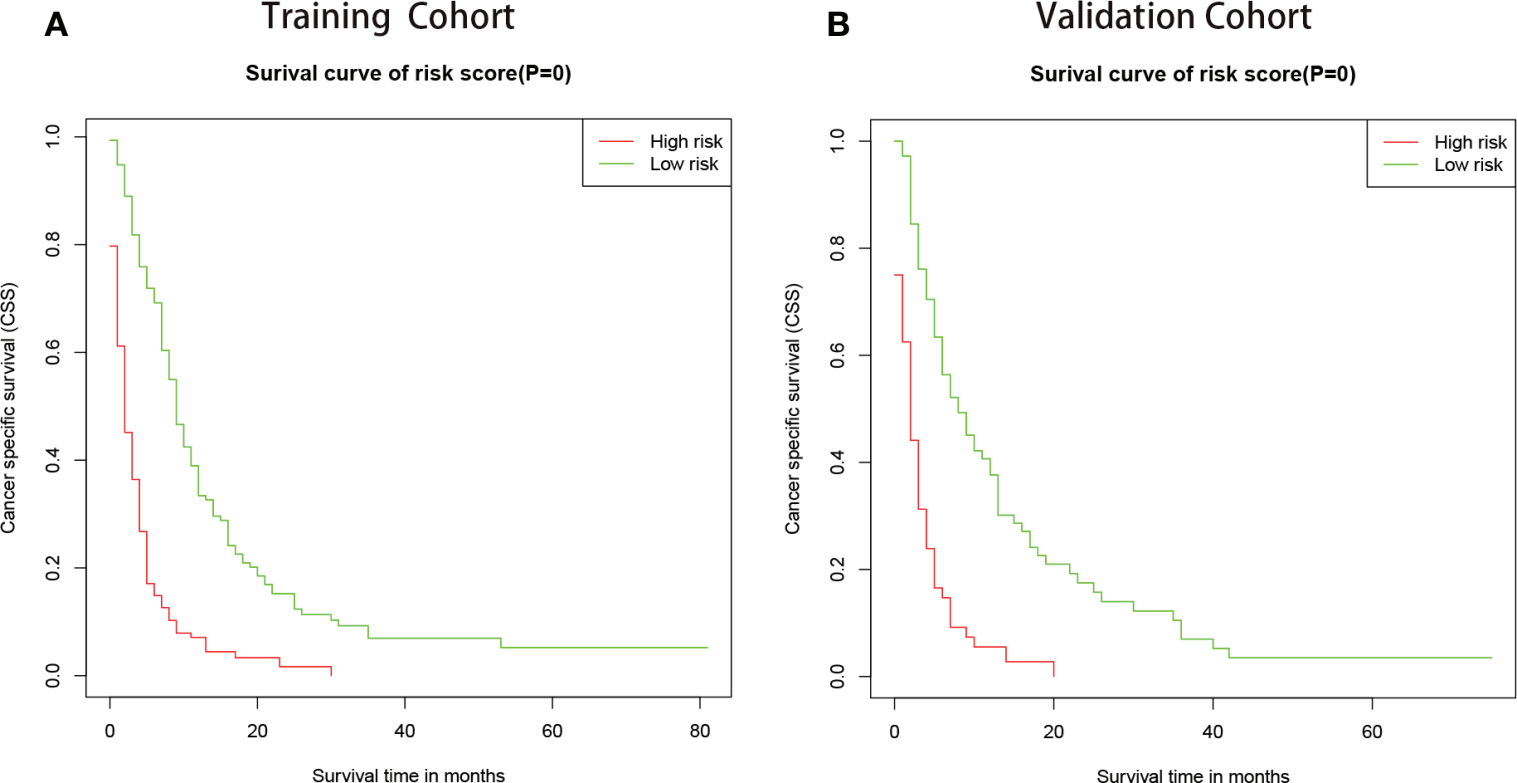
Kaplan–Meier curves of CSS for patients in the low- and high-risk groups. The patients were separated into risk-subgroups according to Cox regression model. **(A)** Kaplan–Meier curves of CSS according to the training cohort. **(B)** Kaplan–Meier CSS curves according to the validation cohort.

To further evaluate the model, we utilized a risk classification, assessment for patients with different CSS. This system in light of each patient’s total risk scores produced by final prognostic factors to split the patients into high risk (risk scores>median) group and low risk (risk scores<median) group. Then, Kaplan-Meier analysis of prognostic curves was performed in both the training cohort (**Figure 6A**) and the validation cohort (**Figure 6B**), which indicated that the CSS among the high- and low-risk groups was differentiated.

## Discussion

The attention of EC patients with synchronous PM has increased in previous years, though these patients contributed only a few percent to EC (13). Due to the limited response to local radiotherapy and chemotherapy, the prognosis of EC patients with synchronous PM is extremely unfavorable. Thus, an effective prognosis predictor is of utmost importance for the optimal management of these patients. However, the prognosis for this patient group cannot yet be properly determined by any assessment technique. Hence, the nomogram, an intuitive statistical forecasting tool, is utilized to evaluate the advanced EC patient’s prognosis and CSS rate with visualization results.

Our study selected 431 cases of EC with a synchronous PM from the SEER database. Based on the results of multivariate Cox regression analyses, the variables (including T stage, liver metastasis, bone metastasis, and chemotherapy) were identified as independent prognostic factors. Next, four factors were taken into account to construct the nomogram that could accurately guide subsequent treatment according to precise predictions of CSS. Additionally, the nomogram model indicated excellent consistency for predicting the 6-, 12- and 18-month CSS via the ROC curve and the calibration curve verification in EC patients with synchronous PM. Moreover, the results of the risk classification assessment showed that the high-risk group portends worse CSS than the low-risk group.

In this study, it is not difficult to see that EC patients with synchronous PM had a poor overall prognosis, similar to the preceding report (14). EC Patients with bone metastases would have a worse CSS than those without. Similarly, the same outcome happened to EC patients with liver metastasis. In particular, EC patients with liver metastases scored higher in the CSS model compared to the patients with synchronous bone metastases. It has been amply proven in multiple prior studies that chemotherapy improves the prognosis of patients with advanced EC (15–17). Patients who did not get chemotherapy had the highest score in the nomogram model, spanning the whole axis. It implies that chemotherapy could increase patients’ probability of surviving.

According to the AJCC Cancer Staging Manual, 7th edition, T1 staging denotes a limited invasion of esophageal cancer that is contained to the mucosa and submucosa. In fact, this limited-stage esophageal cancer is easily overlooked by patients. Tumors that expand more slowly locally tend to have a longer time to progression and thus more accessible to blood vessels. Furthermore, the submucosa, as we know, is rich in blood vessels, and distant metastasis of esophageal cancer is achieved by hematogenous metastasis. As a result, in the population of patients with primary diagnosis of metastatic esophageal cancer, the number of T1 stage may exceed other stages. Additionally, lymphatic metastasis and hematogenous metastasis are two distinct modes of metastasis in esophageal cancer. By reviewing the relevant literatures, we found that there seems to be a subtle association between lymph node metastasis and hematogenous metastasis. For example, older melanoma patients have lower rates of sentinel lymph node metastases yet paradoxically have inferior survival. In vivo, reconstitution of HAPLN1 in aged mice increased the number of LN metastases, but reduced visceral metastases (18). Similarly, androgen receptor increases hematogenous metastasis yet decreases lymphatic metastasis of renal cell carcinoma (19). Besides, patients with larger tumors were more likely to have lymph node metastases (20). Since the majority of the patients in this study were T1 stage and had tiny initial tumors, there were few lymph node metastases. Based on these studies, we suggest that initially diagnosed esophageal cancer with distant metastasis may be more prone to develop fewer lymph node metastases (N0 and N1).

Notably, advanced EC patients with stage T1 have a noticeably worse CSS than patients with stage T2 or T3. And stage T4 has the worst prognosis as cancer of the esophagus invades the peri-esophageal tissues. This could probably be due to the symptoms of the EC of stage T1 are not obvious and the disease has entered a more serious stage when pulmonary metastases occur. Therefore, compared to stage T2 and T3, EC patients with stage T1 have significantly adverse CSS in the metastatic EC. For instance, a patient with bone metastasis (20 points), liver metastasis (30 points), T1 stage (62.5 points), and chemotherapy (0 points) result in the estimated 6-, 12- and 18-month CSS of 40%, 10%, and 0% in our study. Getting a total of fifty points, the same patient with stage T2 has estimated the 6-, 12- and 18-month CSS around 75%, 50%, and 30%. In a word, the patient with stage T1 has a remarkably poor outcome, which could result in an increased focus on early cancer metastases. Due to the invasion of para-esophageal tissues, stage T4 EC is related to the poorest CSS than other stages.

In addition, the result of univariate Cox analysis indicated that tumor grade (P<0.05) was an independent prognostic factor on the CSS of patients. Although tumor grade proved to be prognostic factors independent of metastatic EC (21, 22), classification in the studies were either inelegant or small in the sample size. Furthermore, the pathological tissue sampling site of advanced EC patients may result in differences in tumor grade classification due to the intra-tumor heterogeneity (23, 24). Given that the completeness and applicability of the pathological samples could not be assessed, we did not include the factor of tumor grade in our predictive model. Besides, the univariate analysis also showed that radiotherapy had a significant effect on patient survival. Palliative radiotherapy can relieve the local symptom of advanced EC patients, but there is no statistically significant effect of radiotherapy on overall survival (25). Interestingly, a study of radiotherapy for overall survival and CSS in metastatic esophageal cancer suggested that esophageal squamous cell carcinoma could obtain survival benefit from radiotherapy (P<0.01), but esophageal adenocarcinoma reached the opposite conclusion (26). Indeed, the pathological type of adenocarcinoma was present in more than fifty percent of our study. Since the frequency, dosage, periods, and more details of radiotherapy cannot be acquired from the SEER database, we excluded this factor from the model. Similarly, brain metastasis (P<0.05) seemed to be an independent factor affecting patients’ prognosis based on the univariate Cox regression analysis. However, only less than 7% of EC patients diagnosed with brain metastasis in our training cohort, which is a significant reason why we did not include this factor in our final model.

The finding of this study revealed that chemotherapy had a remarkable influence on the CSS of EC patients with synchronous PM. However, there was no comprehensive information on chemotherapy in the SEER database. At the same time, the lack of population data from different countries may hinder the widespread application of this predictive model.

## Conclusions

A nomogram model was established to accurately assess the prognosis and the CSS of EC patients with synchronous PM. The model contained three clinical factors and a treatment factor and performed well on both training and validation cohorts. It would be utilized to estimate the individualized CSS and guide therapeutic decisions. Palliative chemotherapy presented in the model could improve the CSS of the EC patients with synchronous PM.

## Data availability statement

Publicly available datasets were analyzed in this study. This data can be found here: SEER database.

## Author contributions

C-LZ conceived and designed the study with X-YZ and Q-YL. X-YZ and Q-YL collected the data, drafted the manuscript, analyzed the data and formatted the images and the article. C-LZ reviewed the data. All authors read and approved the final manuscript. All authors contributed to the article and approved the submitted version.
